# A case-control study of musculoskeletal impairment: association with socio-economic status, time use and quality of life in post-conflict Myanmar

**DOI:** 10.1186/s12889-019-7851-5

**Published:** 2019-11-11

**Authors:** Islay Mactaggart, Nay Soe Maung, Cho Thet Khaing, Hannah Kuper, Karl Blanchet

**Affiliations:** 1 International Centre for Evidence in Disability, London School of Hygiene & Tropical Medicine, Keppel Street, London, WC1E 7HT UK; 2 Health in Humanitarian Crises Centre, London School of Hygiene & Tropical Medicine, Keppel Street, London, WC1E 7HT UK; 3 University of Public Health (UPH), Myorma Kyaung Street, Yangon, Myanmar

**Keywords:** Musculoskeletal impairment, Physical rehabilitation, Quality of life, Myanmar

## Abstract

**Background:**

Musculoskeletal impairments (MSI) are a major global contributor to disability. Evidence suggests entrenched cyclical links between disability and poverty, although few data are available on the link of poverty with MSI specifically. More data are needed on the association of MSI with functioning, socio-economic status and quality of life, particularly in resource-poor settings where MSI is common.

**Methods:**

We undertook a case-control study of the association between MSI and poverty, time use and quality of life in post-conflict Myanmar. Cases were recruited from two physical rehabilitation service-centres, prior to the receipt of any services. One age- (+/− 5 years of case’s age) and sex- matched control was recruited per case, from their home community. 108 cases and 104 controls were recruited between July – December 2015. Cases and controls underwent in-depth structured interviews and functional performance tests at multiple time points over a twelve-month period. The baseline characteristics of cases and controls are reported in this manuscript, using multivariate logistic regression analysis and various tests of association.

**Results:**

89% of cases were male, 93% were lower limb amputees, and the vast majority had acquired MSI in adulthood. 69% were not working compared with 6% of controls (Odds Ratio 27.4, 95% Confidence Interval 10.6–70.7). Overall income, expenditure and assets were similar between cases and controls, with three-quarters of both living below the international LMIC poverty line. However, cases’ health expenditure was significantly higher than controls’ and associated with catastrophic health expenditure and an income gap for one fifth and two thirds of cases respectively. Quality of life scores were lower for cases than controls overall and in each sub-category of quality of life, and cases were far less likely to have participated in productive work the previous day than controls.

**Conclusion:**

Adults with MSI in Myanmar who are not in receipt of rehabilitative services may be at increased risk of poverty and lower quality of life in relation to increased health needs and limited opportunities to participate in productive work. This study highlights the need for more comprehensive and appropriate support to persons with physical impairments in Myanmar.

## Background

One billion people, or 15% of the global population, is estimated to have a disability – 80% of whom live in low and middle income country (LMIC) settings [1].

Musculoskeletal impairments (MSI) – namely those that affect the physical functions, movements and structure of a person’s body – are a major contributor to disability globally [2, 3]. Musculoskeletal impairments include impaired functions, movements or structure of the joints, bones and muscles, and can be congenital, neurological or acquired through illness, injury or trauma [4]. The global magnitude of MSI is unknown, in part due to the heterogeneity of conditions the term encompasses. However, studies conducted in Rwanda, Cameroon and India between 2005 and 2014 estimated the prevalence to vary between 3.4–5.2% of the all-age population, increasing substantially with age [4, 5].

A growing body of evidence has identified pervasive cyclical links between disability and lower socio-economic status (SES), particularly in LMICs [6]. Having a disability is associated with more frequent health risks, and consequently greater risk of catastrophic health costs, exacerbating poverty [1, 7–9]. Persons with disabilities also face greater barriers to work, leading to higher unemployment rates and lower SES of people with disabilities and their households [10–12]. Conversely, poverty can heighten the risk of disability through exclusion from health, rehabilitative and other services; increased exposure to risk factors for poor health; and heightened environmental risks, such as from unsafe work environments [13–15].

Few data are available that disaggregate the relationship between disability and poverty by impairment type. One recent survey of MSI in Rwanda established that adults with MSI were over three times less likely to be working than adults without, but did not find differences in terms of SES [16]. Moreover, limited data are available on the relationship between disability and time use, particularly by impairment type, which may help to unravel the relationship of disability with poverty and quality of life.

In 2011, the Republic of the Union of Myanmar elected a civilian government following fifty years of military rule [17]. It is one of a small number of countries globally with continually high rates of landmine causalities, following lengthy conflict [18]. In addition, unintentional injury related to road accidents, falls and mechanical force injuries remain common in the country, and are leading causes of MSI [19].

More data are urgently needed on the relationship between MSI, poverty, and the effect of physical rehabilitation on quality of life in LMIC settings. This information is particularly needed in conflict and post-conflict affected settings, where increased risk of trauma and injury heighten the magnitude of MSI [20]. This study therefore set out to assess the link between MSI with poverty, quality of life, and time use in Myanmar.

## Methods

### Study overview

We undertook a case-control study of the association between MSI and poverty, time use and quality of life in post-conflict Myanmar. Cases were recruited from two physical rehabilitation centres, prior to receipt of rehabilitation services. One age- (+/− five years of the case’s age) and sex- matched control was recruited per case, living in the same community as the case and having no physical impairment. All cases and controls underwent in-depth interview using a structured questionnaire.

### Sample size calculation

There is no existing data on the possible association between MSI and poverty on which to calculate a case-control study design sample size [21]. Consequently, this study followed Norman et al.’s recommendation that a sample size of 64 per group would detect a medium effect size of 0.5 [22]. Accounting for prospective drop out of up to 40% at one year post follow up, a sample of 100 cases and 100 controls was recruited.

### Participant recruitment

Cases were recruited from two physical rehabilitation centres in Myanmar, which were the main providers of prostheses and orthoses in the country in 2015: The National Rehabilitation Hospital in Yangon (NRH, operated by the Myanmar Ministry of Health), and the Hpa-An Orthopaedic and Rehabilitation Centre in Hpa-An (HORC, operated by the Myanmar Red Cross Society in collaboration with the Ministry of Health and the International Committee of the Red Cross).

Centre clients were eligible for enrolment in the study if they:

- were ≥ 18 years old,
- had never previously been fitted with a prosthetic or orthotic assistive device,
- were determined by a trained physiotherapist to require either a prosthetic or orthotic device due to MSI,
- were able to communicate independently or via translator,
- did not plan to migrate outside of Myanmar within the following twelve months.

Clients at NRH and HORC meeting the above criteria were provided oral and written information about the study and requested to formally consent to participate. All clients were assured that they had the right not to participate and that this would not affect the services they received.

For each client who met the eligibility criteria and agreed to participate (“cases”), one matched control was identified from the same local community as the case. Controls were identified as follows: the same sex as the case, +/− five years of age, able to communicate independently or via translator, not planning to migrate outside of Myanmar within the following twelve months and did not have an MSI.

To identify controls, data collectors accompanied cases to their homes or were provided with information from the cases to identify their home independently. The data collector spun a bottle outside the case’s house and walked in the direction of the bottle to the nearest house to identify a control matching the above criteria. If an eligible control was available, the data collector provided relevant study information and asked the control if they wished to participate before taking written consent and beginning the interview.

If no eligible control was identified within the household, or the eligible control chose not to participate, the data-collector returned to the case’s household, re-spun the bottle and continued the process until an eligible control was identified.

### Data collection

Cases were assessed using the Rapid Assessment of Musculoskeletal Impairment (RAM) tool to identify MSI presence, severity and aetiology according to pre-validated algorithms [4, 23]. The RAM was developed and validated for use in LMICs, and has been previously used in Kenya, Rwanda, Cameroon and India [23–25].

Physical functioning was assessed using two standardised tools: the Physical Performance Test (PPT) and the Two Minute Walk Test (TMWT [26]. The PPT comprises nine items, scoring an individual based on the time it takes them to complete each task. A score of 0 relates to inability to complete a task, with higher scores for quicker completion rates. The PPT has not previously been used in low income settings. The TMWT is a widely validated test of aerobic capacity and endurance in post-stroke rehabilitation, spinal cord injury and amputation [27–29]. The TMWT measures the distance ambulated in two minutes on flat ground.

Time use was measured using the ‘Stylised Activity List’ developed by the Living Standards Measurement Study [30]. The tool contains thirteen broad activities comprising areas of personal care (e.g. sleeping, bathing/dressing and medical care), productive activities (both paid and non-paid activities including household tasks), leisure (in and outside the household) and time spent resting (no activity). The number of hours spent undertaking each activity on the previous day is recorded, alongside whether or not assistance was needed. This tool has previously been used in assessing the long term impact of cataract surgery in Bangladesh, Philippines and Kenya [31].

We also used the WHOQOL-BREF, developed by the World Health Organisation (WHO) to assess quality of life. The WHOQOL-BREF comprises 26 items related to physical, psychological, social and environmental domains of quality of life, and uses Likert scale responses ranging between very poor/very dissatisfied/not at all, and very good/very satisfied/an extreme amount. The WHOQOL-BREF has shown excellent reliability and validity in more than 20 countries [32].

SES was measured in three different ways, each in accordance with World Bank recommendations of reliable and comparable collection of household SES data in LMICs [33]: (i) Household income was measured directly as reported average monthly income in the household; (ii) Household expenditure was measured across 85 pre-validated, pilot-tested items related to expenditure on food (including value via home production, received in kind or as gifts), education, health, household and personal items and rent [34]; and (iii) Asset ownership was measured using a pre-tested asset list (33 items) to assess the number and type of assets owned by the household (e.g. furniture, vehicles, cattle) and key characteristics of the household structure (e.g. building materials, number of rooms).

All questions related to socio-economic status were asked directly to the person in the household with primary responsibility for the household’s finances.

### Training and field work

Mid-level rehabilitation professionals (e.g. orthopaedic technicians, physiotherapists or physiotherapist assistants) at NRH and HORC were provided training to assist data collection through recruitment and physical assessment of eligible clients.

In addition, six full-time data collectors were recruited from local universities. A two-week training course was held in July 2015 incorporating modules on disability sensitisation (led by a local disabled persons’ organisation), project protocol and data collection tools, informed consent and ethics, study logistics and recruitment, safety and security.

Ten volunteers were recruited from NRH, alongside ten community volunteers as part of the training programme, to pilot-test the tools and study approach. Data was collected, stored and managed using a bespoke Android application, built using Python coding and deployed using Google Nexus tablets.

### Statistical analysis

Data were cleaned and analysed in Stata 14.0 [35]. Perfect matching between cases and controls was not achieved, excluding paired analysis approaches.

Chi-squared tests of association and age-sex adjusted logistic regression analyses were used to measure differences in socio-demographic characteristics between cases and controls, whilst descriptive statistics were used to describe case service-centre details.

PPT scores were divided into categories based on crude thirds (0–12, 13–24 and 25–36). PPT category and TMWT average distance were compared between cases and controls using Chi-squared and student t-tests of association/difference respectively.

Household monthly income was divided by household size to estimate Per Capita Income (PCY). Similarly, Personal Consumption Expenditure (PCE) was calculated by dividing household expenditure by household size. Both PCY and PCE were converted into US dollars for ease of interpretation. The assets list was used to derive a household-level relative index indicating SES, via Principle Components Analysis (PCA) and categorised into tertiles [36]. PCA involves a statistical calculation of the relative weight of different assets, producing a total score per household.

Due to the skewed nature of income and expenditure variables, raw PCE and PCY results were logged, and exponentiated regression coefficients were derived using linear regression, accounting for age and sex. Age-Sex adjusted Logistic Regression was used to derive odds ratios for the proportion of cases and controls experiencing catastrophic health expenditure (≥ 10% monthly per capita expenditure [37]), below the international Lower Middle Income Country Poverty Line (3.20 USD, adjusted for Purchasing Power Parity), in each PCA tertile, and experiencing an income gap (PCE > PCI).

Time-use allocation was aggregated and any responses totalling less than 19 or greater than 29 h were removed from the analysis. Age-sex adjusted logistic regression was used to compare participation in different activities amongst cases and controls. Logged linear regression was undertaken, accounting for age and sex, to assess differences in the proportion of time spent in different activities between cases and controls.

Quality of Life scores were aggregated and transformed into scores out of 100. Mean scores were compared using a student t-test.

Multivariate logistic regression analyses were undertaken amongst cases to ascertain associations between:

i) case quality of life scores (general quality of life score, general health quality of life score, physical heath quality of life score and psychological health quality of life score respectively)
ii) and age group, work status, proportion of the day spent resting, proportion of the day spent in productive activities, physical functioning score, PCA tertile, PCE quartile, PCI quartile and proportion experiencing income gap.

### Ethical approval

Ethical approval for the study was granted by the Observational Research Ethics Committee (ref 9292) at the London School of Hygiene & Tropical Medicine and the Myanmar Ministry of Health Ethical Review Board (Ref 1/2015).

## Results

Table 1 presents the socio-demographic characteristics of study participants. 108 cases were recruited, alongside 104 controls. Cases and controls were well matched on age and gender, although 89% of each were male.

**Table 1:** Socio-demographics of study participants

		Cases (*n* = 108)	Controls (*n* = 104)	Age-Sex adj OR (95% CI)
		%	%	
Gender	Male	89%	89%	Ref.¤
	Female	11%	11%	1.0 (0.4–2.5)
Age	18–39	43%	42%	Ref.
	40–59	44%	44%	1.1 (0.5–1.7)
	60+	14%	13%	1.0 (0.5–1.7)
Marital Status	Married	64%	72%	Ref.
	Not Married	36%	28%	1.6 (0.9–2.9)
Religion	Theravada Buddhism	90%	93%	1.6 (0.6–4.3)
	Other	10%	6%	Ref.
Ethnicity	Bamar	76%	80%	Ref.
	Kayin	12%	10%	1.3 (0.6–3.2)
	Rakhine	6%	6%	1.0 (0.3–3.3)
	Other	7%	5%	1.4 (0.4–4.6)
Literacy	Reads well	77%	88%	Ref.
	Reads a little	16%	10%	1.9 (0.8–4.5)
	Does not read at all	7%	3%	3.1 (0.8–12.1)
Employment status	In the field only	5%	36%	0.3 (0.1–0.8)
	Job other than in the field	26%%	59%	Ref.
	No job and no work in the field	69%	6%	27.4 (10.6–70.7)
Head of household	Yes	57%	74%	Ref.
	No	43%	26%	3.7 (1.7–8.0)

¤Denotes reference group in odds ratio calculation throughout

There were no differences between cases and control in marital status, religion, ethnicity or literacy. However, cases were more likely than controls not to be working (69% versus 6%, Odds Ratio 27.4, 95% confidence interval 10.6–70.7), and were also less likely to be the head of their household (57% versus 74%, OR 3.7, 95% CI 1.7–8.0).

Approximately half of the cases were recruited at each of the two sites (43% at HORC, 58% at NRH, Table 2). Only 2% reported that they had acquired MSI congenitally or in the first 15 years of life, with 46% acquired trauma and 23% related to non-acquired trauma. 98% of cases were lower limb amputees and over 90% were assessed to require an above or below knee prosthetic. (See Table 2).

**Table 2:** MSI-related information among cases

		%
Service Centre	Red Cross Centre (HORC)	43%
	National Rehabilitation Centre (NRH)	58%
Age at which MSI acquired	Since birth	1%
	0–15 years old	1%
	16–39 years old	46%
	40+	52%
Origin of MSI	Congenital/ Genetic	2%
	Infection	10%
	Acquired Trauma	56%
	Neurological	8%
	Acquired Non Traumatic	23%
Amputee	Yes	98%
	No	2%
Device to be fitted	Below Knee (BK) prosthetic	53%
	Above Knee (AK) prosthetic	40%
	Knee Disarticulation (KD) prosthetic	4%
	Above Elbow (AE) prosthetic	3%
	Orthosis (AFO, KAFO or Milwaukee)	2%

Table 3 describes baseline physical functioning information for cases and controls. 100% of controls were categorised in the highest tertile of physical performance using the Physical Performance Test (PPT), compared with 44% of cases (*p* < 0.001). 95% of cases used an assistive device to perform the Two Minute Walk Test (TMWT). On average, cases were able to walk 65.6 m in two minutes (standard deviation 29.5), compared with 133.5 m on average for controls (sd 102.7, *p* < 0.001).

**Table 3:** Physical Functioning information

		Cases (*n* = 108)	Controls (*n* = 104)	*P*-value (χ^2^)
Physical Performance Test (PPT)	0–12 (lowest)	10%	0	< 0.001
	13–24	46%	0	
	25–36 (highest)	44%	100%	
	Mean score	22.6	33.1	< 0.001
	Standard deviation (sd.)	6.3	2.0	< 0.001
Two Minute Walk Test (metres)	0	5%	0	< 0.001
	1–50	24%	4%	
	51–100	62%	57%	
	101–200	9%	25%	
	201+	0	14%	
	Uses Assistive Device	95%	0	< 0.001
	Mean Distance	65.6	133.5	< 0.001
	Standard deviation (sd.)	29.5	102.7	

Socio-economic status is described in Table 4. There were no differences between cases and controls in overall per capita expenditure. Per capita expenditure on health care was significantly higher amongst cases compared to controls (a median monthly per capita expenditure of $0.18 for cases compared with zero for controls) but there was no difference in per capita expenditure for the other categories. There were no differences in median per capita income between cases and controls, nor in the proportion of cases and controls below the international poverty line or the poorest per capita income quartile. However, cases were much more likely to experience catastrophic health expenditure (20.4% versus 1.9%, OR 15.2, 95% CI 3.3–69.8) and more likely to experience an income gap (65.7% versus 47.1%, 2.2, 1.2–3.8) than controls.

**Table 4:** Socioeconomic status

		Cases (*n* = 108)		Controls (*n* = 104)		Coefficient (95% CI)^a^
		Median (95% CI) ^a^	(IQR) ^a^	Median (95% CI) ^a^	(IQR) ^a^	
Per Capita Expenditure (US$, monthly)	Total	35.03	34.72	32.07	35.50	11.8% (−8.6–32.2)
	Food	18.28	14.76	18.15	15.69	−6.0% (−21.0–8.9)
	Health	0.18	2.36	0.00	0.47	115.5% (51.3–179.8)
	Other^b^	12.57	19.18	11.52	17.72	9.2% (−19.5–38.0)
	Total minus health	32.41	27.14	31.06	33.54	2.2% (−16.9–21.2)
Catastrophic Health Expenditure		20.4%		1.9%		15.2 (3.3–69.8)
Below International Poverty line		75.0%		76.9%		0.9 (0.5–1.7)
Per Capita Income (US$, monthly)		3.1	0.8	3.4	0.9	−19.5% (−39.5–0.6)
Quartile 1 (poorest)		38.9%		26.2%		Ref.
Quartile 2		25.0%		25.2%		0.7 (0.3–1.4)
Quartile 3		15.7%		24.3%		0.4 (0.2–1.0)
Quartile 4 (richest)		20.4%		24.3%		0.6 (0.3–1.2)
Proportion experiencing an income gap		65.7%		47.1%		2.2 (1.2–3.8)^c^
SES score	SES score	−0.2 (−0.7–0.2)	2.5	0.2 (−0.2–0.7)	2.4	
						Age-Sex adj OR (95% CI)
	Tertile 1 (poorest)	42.6%		33.7%		0.6 (0.3–1.2)
	Tertile 2 (middle)	29.6%		33.7%		
	Tertile 3 (richest)	27.8%		32.7%		

^a^Exponentiated regression coefficient, using log transformed expenditure and income, which illustrates the percent difference in expenditure/income per capita among case and control households, after taking into account age and sex

^b^Other expenditure: personal, entertainment, education, taxes, occasional and other

^c^Amongst those reporting an income gap < 0

Cases were much more likely to have allocated time to medical care in the previous twenty four hours than controls (88.5% versus 27.2%, 21.1, 10.0–44.8), and much less likely to have allocated time for either household, paid or non-paid work than controls in the previous 24 h (Table 5). Median amount of time spent working the previous day was zero minutes amongst cases and six hours for controls, meaning that cases had spent 33.5% (95% CI 5.3–61.8%) less time on household work, and 88.5% (48.7–128.2%) less time on paid/non-paid work than controls in the previous 24 h.

**Table 5:** Time Use among cases and controls

	Cases (*n* = 104) (%)^b^		Controls (*n* = 103) (%)^b^		Age-Sex adj OR (95% CI)
*Participation*					
Personal/self care	100%		100%		–
Medical care	88.5%		27.2%		21.1 (10.0–44.8)
Household Work	58.7%		72.8%		0.5 (0.3–0.9)
Paid Work or Work for Own Use	17.3%		91.3%		0.02 (0.01–0.4)
Leisure out of the home	16.3%		33.0%		0.4 (0.2–0.8)
Leisure in the home	98.1%		98.1%		1.0 (0.1–7.4)
No specific activity	99.0%		95.2%		5.0 (0.5–46.1)
	Median (95% CI)	(IQR)	Median (95% CI)	(IQR)	Coefficient (95% CI)^a^
*Proportion of time*					
Personal/self-care	10:05	01:40	09:30	01:30	4.5% (0.6–9.3)
Medical care	03:00	04:00	0	00:15	90.6% (51.9–129.3)
Household Work	00:15	00:30	00:30	02:00	−33.5% (−61.8 – −5.3)
Paid Work or Work for Own Use	0	0	06:00	04:30	−88.5% (−128.2 – −48.7)
Leisure out of the home	02:30	02:45	04:00	00:30	−40.7% (− 168.7–87.2)
Leisure in the home	04:00	01:30	03:00	02:00	39.6% (26.6–52.6)
No specific activity	04:00	02:00	02:00	03:00	52.3% (25.0–69.6)

^a^Exponentiated regression coefficient, using log transformed time use in minutes, which illustrates the percent difference in time spent on activities the previous day after taking into account age and sex

^b^Time Use data was removed for four cases and one control due to missing values

**Fig. 1:**
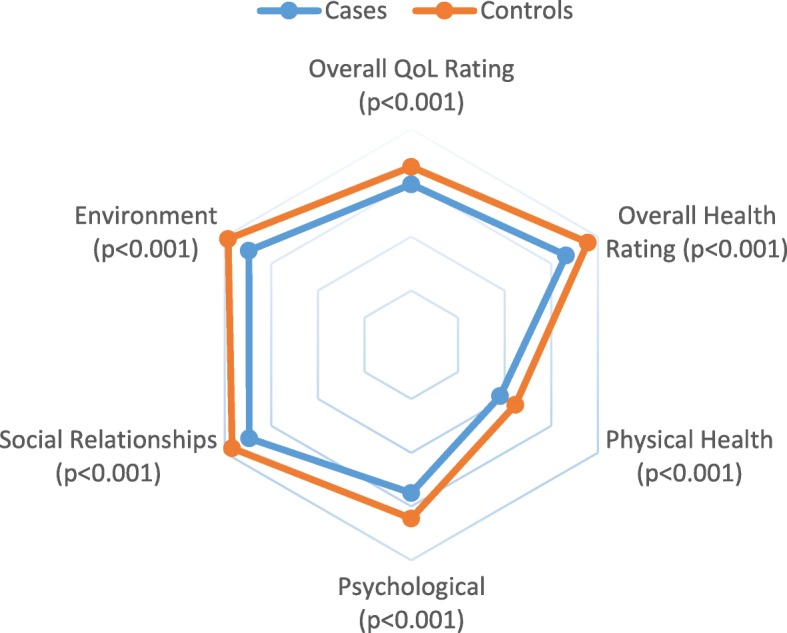
Baseline Quality of Life

Quality of life scores were lower for cases than controls overall and for each category of quality of life (Table 6, Fig. 1). The difference between mean scores was greatest in the domains of general (9.4, *p* < 0.001) and psychological (9.4, *p* < 0.001) health. Multivariate logistic regression was undertaken to explore predictors of low quality of life amongst cases (Table 7). Lower general health and lower psychological quality of life were noted amongst cases that were not working.

**Table 6:** Quality of life

	Cases (*n* = 108)		Controls (*n* = 104)		Difference between means	*P*-value^a^
	Mean (95% CI)	SD	Mean (95% CI)	SD		
General QoL Rating	59.6 (56.6–62.7)	16.0	66.0 (63.5–68.4)	12.4	6.4	< 0.001
General Health Rating	66.4 (52.5–70.2)	20.3	75.8 (72.9–78.7)	14.9	9.4	< 0.001
Physical Health	38.1 (36.1–40.1)	10.4	44.7 (42.5–46.9)	11.3	6.6	< 0.001
Psychological Health	55.1 (52.6–57.6)	13.0	64.5 (62.6–66.5)	10.1	9.4	< 0.001
Social Relationships	69.5 (67.0–72.1)	13.4	76.9 (75.9–78.8)	10.2	7.4	< 0.001
Environment	69.7 (66.9–72.4)	14.4	78.6 (76.1–81.0)	12.5	8.9	< 0.001

^a^Student T Test

**Table 7:** Quality of life MVA

		General QoL Rating	General Health	Physical Health	Psychological Health
		Average Score (95% CI)	Average Score (95% CI)	Average Score (95% CI)	Average Score (95% CI)
Age Group	18–49	57.6 (53.7–61.6)	64.7 (59.7–69.7)	37.7 (35.3–40.0)	56.0 (52.9–59.0)
	50+	63.1 (58.2–67.9)	69.2 (62.9–75.5)	38.9 (35.1–42.6)	53.7 (49.2–58.1)
	p for trend	0.3	0.3	0.7	0.5
Work	Yes	61.2 (56.2–66.2)	73.3 (67.3–79.4)	36.9 (34.3–39.5)	59.5 (55.7–63.3)
	No	58.9 (55.0–62.8)	63.2 (58.4–68.1)	40.9 (38.4–43.5)	53.1 (50.1–56.2)
	p for trend	0.6	< 0.05	0.1	< 0.05
Proportion of day spent resting (no activity)	Lowest	62.2 (56.8–67.6)	66.5 (59.8–73.2)	36.9 (34.1–39.7)	57.6 (53.9–61.2)
	Middle	57.1 (51.3–62.9)	68.0 (61.5–74.5)	39.3 (35.6–43.1)	53.5 (49.9–57.1)
	Highest	59.4 (54.3–64.6)	64.6 (56.9–72.2)	39.3 (35.8–42.8)	55.7 (50.9–60.5)
	p for trend	0.5	0.8	0.2	0.5
Proportion of day spent in productive activities	Lowest	58.4 (53.6–61.2)	67.3 (61.5–73.2)	39.1 (36.1–42.1)	56.1 (52.5–59.8)
	Middle	55.8 (58.7–69.5)	62.5 (54.5–70.5)	36.8 (32.2–41.3)	50.0 (46.4–53.6)
	Highest	61.1 (58.7–69.5)	67.4 (60.2–75.1)	38.8 (35.9–41.6)	58.8 (54.8–62.9)
	p for trend	1.0	1.0	0.8	0.4
Physical Functioning Score	Lowest	52.7 (43.7–61.8)	60.0 (45.3–74.7)	36.5 (31.2–41.9)	51.9 (41.2–62.4)
	Middle	59.2 (54.9–63.5)	64.0 (58.1–69.9)	38.6 (35.9–41.2)	55.5 (52.2–58.8)
	Highest	61.7 (56.6–66.9)	70.4 (64.7–76.1)	38.0 (34.4–41.5)	55.3 (51.4–59.5)
	p for trend	0.1	0.06	0.8	0.4
PCA^a^ Tertile	Lowest	57.4 (52.1–62.6)	65.2 (59.3–71.1)	37.9 (34.9–40.9)	54.3 (49.9–58.8)
	Middle	60.6 (55.1–66.2)	67.7 (61.0–74.5)	39.1 (36.5–41.8)	57.4 (53.7–61.0)
	Highest	62.0 (56.7–67.3)	66.7 (58.0–75.3)	37.4 (32.4–42.5)	54.0 (49.5–58.5)
	p for trend	0.19	0.7	0.5	0.7
PCE^b^ Quartile	Q1 (Lowest)	49.6 (42.3–56.9)	65.2 (56.5–74.0)	36.0 (31.8–40.3)	48.7 (44.4–52.9)
	Q2	64.6 (58.4–70.8)	68.5 (60.8–76.1)	35.0 (31.0–39.0)	55.2 (49.3–61.1)
	Q3	61.9 (56.5–67.4)	60.0 (52.7–67.3)	39.0 (35.7–42.1)	55.6 (50.7–60.6)
	Q4 (Highest)	60.7 (55.2–66.3)	72.6 (64.3–80.8)	42.1 (37.3–46.8)	59.8 (55.6–64.0)
	p for trend	< 0.05	0.5	< 0.05	< 0.01
PCI^c^ Quartile	Q1 (Lowest)	57.1 (52.1–62.2)	61.9 (55.8–68.0)	37.7 (35.3–40.2)	53.4 (49.9–56.8)
	Q2	60.8 (54.1–67.4)	66.2 (58.3–74.0)	36.0 (30.9–41.1)	54.7 (47.7–61.7)
	Q3	56.5 (49.9–63.0)	64.7 (52.9–76.5)	37.8 (33.4–42.2)	57.1 (51.4–62.7)
	Q4 (Highest)	65.5 (58.1–72.8)	76.4 (68.3–84.4)	41.6 (36.2–47.1)	57.4 (52.4–62.5)
	p for trend	0.14	< 0.05	0.3	0.09
Income Gap	Yes	60.0 (56.34–63.7)	68.9 (62.3–75.4)	38.6 (36.5–40.7)	54.0 (49.0–58.9)
	No	58.9 (2.9–64.7)	65.1 (60.2–70.0)	37.2 (32.8–41.5)	55.7 (52.9–58.5)
	(χ^2^)	0.3	0.7	0.3	0.6
Model *P* value		< 0.001	0.14	0.2	< 0.01
Model R^2^		29.8%	21.6%	19.5%	30.0%

^a^Principal Component Analysis; ^b^Per Capita Expenditure; ^c^Per Capita Income

## Discussion

### Summary of key findings

Almost all (89%) cases enrolled in this study were male, and the vast majority had acquired MSI in adulthood. 69% were not working at the time of enrolment (compared with 6% of controls), and 93% were amputees requiring below or above knee prosthetics and substantial physical rehabilitation.

Three quarters of both cases and controls lived below the international LMIC poverty line of $3.20 per person per day. However, cases’ expenditure on health was significantly higher than controls’, and associated with catastrophic health expenditure and an income gap for one fifth and two thirds of cases respectively.

Cases were far less likely to have participated in work (either housework or paid/non-paid work) than controls the previous day, and the median proportion of time spent in productive activities was lower. Cases instead spent significantly more time engaged in medical care compared with controls (3 h versus zero hours) and in resting with no specific activity (4 h versus 2 h).

Quality of life scores were lower for cases than controls in each domain, and particularly in terms of general and psychological health.

### Socio-demographics and physical functioning

Most cases reported traumatic and non-traumatic acquisition of MSI in adulthood, suggesting that these were preventable both in terms of the prevention of trauma, and of acquired health conditions resulting in secondary amputation. These findings reinforce calls for the growing burden of - and exclusion related to - MSI in LMICs to be addressed [38]. The limited number of female cases compared to males may be a reflection of the fact that statistically, men are at higher risk of limb loss than women, particularly in cases of traumatic injury [39]. Men may also exhibit higher rates of risk-taking behaviour associated with cardio-vascular conditions. For example, smoking is six times more prevalent among men than women in Myanmar [40]. However there may also be gender disparities in health-seeking behaviour and capacity to access appropriate services that have contributed to this imbalance. Unsurprisingly, persons with MSI in the study had much lower physical functioning compared to controls – highlighting the physical impact of MSI on an individual’s functionality.

### Socio-economic status

A recent systematic literature review reported evidence of a positive association between disability and economic poverty in 81% of the 122 included studies [41]. The review also established that the proportion of studies reporting a positive association was higher amongst middle income countries compared to lower income countries. This suggests that high levels of absolute poverty experienced across the population, as in Myanmar where three quarters of participants lived below the international LMIC poverty line, may mask disparities in relation to impairment.

However, despite similar per capita income and expenditure amongst people with and without MSI, people with MSI were far more likely to experience catastrophic health expenditure and an income gap than people without in the study. This finding reflects the prevailing literature on the “extra costs” of disability that are borne directly by households, often in direct relation to healthcare, and which exacerbate multi-dimensional poverty [42, 43].

Compounding the additional health expenditures related to impairment, the findings additionally highlight the substantial exclusion from income-generation experienced by people with physical impairments in Myanmar. Only 17% of cases reported that they had participated in paid work, or unpaid work otherwise for their own use, in the previous 24 hours, compared with 91.3% of controls. The limited, but expanding, evidence base suggests that barriers to livelihoods are common amongst people with disabilities in LMICs, and in particular for persons with physical impairments [11, 44]. Evidence is needed on effective support and interventions to overcome these barriers, which may include policy change, social protection, health insurance and access to rehabilitation services and appropriate assistive devices [10].

### Time use and quality of life

The implications on wellbeing of the more vulnerable socio-economic situation of persons with physical impairments in the study and their households, compared to matched controls, are further highlighted by time use and quality of life metrics. Whilst the study is cross-sectional in nature, precluding comment on causality, poverty and vulnerability have previously been shown to be associated with poorer quality of life, and poorer mental health in a number of other studies [45]. Moreover, a recent review of psychosocial adjustment to lower-limb amputation emphasised the associations between limb loss, low mood and anxiety, particularly in the initial post-amputation phase (< 2 years) [46]. This may represent an adjustment reaction to limb loss and sudden disability, which subsequently improves [47]. However there can be long lasting problems relating to amputation, including residual limb issues, phantom pain and pressure sores [48], which may increase the likelihood and persistence of depression and anxiety [49].

### Study strengths and limitations

This data is cross-sectional and therefore we are unable to ascribe causality to the associations between MSI, poverty and quality of life identified. This study may potentially have been under-powered for certain stratified analyses. In addition, limited recent literature on the correlates of MSI exist with which to compare and contrast these findings. However, this is the first study to our knowledge to explore the impact of MSI in people’s lives in Myanmar in a comprehensive and systematic way, and as such provides important preliminary evidence in this area.

## Conclusion

This study highlights the negative link between MSI, socio-economic status, time-use and quality of life in post-conflict Myanmar. The interconnectedness of physical functionality, access to livelihoods, socio-economic vulnerability, time use and quality of life are apparent in the study findings, and highlight the need for more comprehensive and appropriate support to persons with physical impairments in Myanmar. In particular, the study highlights the need for further evidence generation of the impact of physical rehabilitation and other services to support inclusion of persons with physical impairments in the country.

## Data Availability

The datasets used and/or analysed during the current study are available from the corresponding author on reasonable request.

## Abbreviations

AE: Above Elbow [Prosthetic]
AFO: Ankle Foot Orthosis
AK: Above Knee [Prosthetic]
BK: Below Knee [Prosthetic]
CI: Confidence Interval
GPS: Global Positioning System
HORC: Hpa-An Rehabilitation Centre
IQR: Inter-Quartile Range
KAFO: Knee Ankle Foot Orthosis
KD: Knee Disarticulation
LMIC: Low or Middle Income Country
MSI: Musculoskeletal Impairment
MVA: Multi-Variable Analysis
NRH: National Rehabilitation Hospital
OR: Odds Ratio
PCA: Principal Component Analysis
PCE: Personal Consumption Expenditure
PCY: Per Capita Income
PPT: Physical Performance Test
RAM: Rapid Assessment of MSI
SD: Standard Deviation
SES: Socio-economic status
TMWT: Two Minute Walk Test
US: United States
WHO: World Health Organisation
WHOQOL-BREF: WHO Quality of Life Brief Tool
